# Experimental Analysis and Establishment of Strength Attenuation Model of POM Fiber Reinforced Geopolymeric Recycled Concrete under Freeze-Thaw Cycles

**DOI:** 10.3390/ma16041699

**Published:** 2023-02-17

**Authors:** Xiaoshuang Shi, Xiaoqi Wang, Qingyuan Wang, Tao Zhang, Fuhua Yang, Yufei Xu, Jinsheng Zhan

**Affiliations:** 1 Key Laboratory of Deep Underground Science and Engineering (Ministry of Education), College of Architecture and Environment, Sichuan University, Chengdu 610065, China; 2 Failure Mechanics & Engineering Disaster Prevention and Mitigation, Key Laboratory of Sichuan Province, Sichuan University, Chengdu 610065, China; 3 CSCEC Southwest Consulting Co., Ltd., Chengdu 610095, China; 4 Southwest Construction Engineering Co., Ltd., China Construction Eighth Engineering Division Co., Ltd., Chengdu 610041, China

**Keywords:** frost resistance, geopolymeric recycled concrete, polyoxymethylene fiber, strength attenuation model, SEM

## Abstract

Geopolymeric recycled concrete (GRC) is a new low-carbon building material that uses both construction and industrial solid waste to replace natural aggregate and cement. GRC is similar to geopolymeric concrete (GPC) in that it has good mechanical properties but needs to be improved in terms of frost resistance. Previous studies have shown that polyoxymethylene fiber (POM fiber) can improve the shrinkage and durability of concrete and is superior to other commonly used fibers. Therefore, this paper explores adding POM fiber to GRC to improve its frost resistance. In this paper, the influence of different volumes and lengths of POM fiber on the frost resistance of geopolymeric recycled concrete (PRGRC) is studied. By measuring the changes in mass loss rate, relative dynamic elastic modulus, and compressive strength of PRGRC under different cycles, the improvement effect of POM fiber on the freeze-thaw damage of GRC is analyzed, and the strength attenuation model of PRGRC is established. The results show that the increase in POM fiber content can effectively slow down the mass loss of PRGRC in the freeze-thaw cycles, the reduction rate of relative dynamic elastic modulus, and the reduction rate of compressive strength. This shows that POM fiber can effectively improve the frost resistance of PRGRC, and the effect of 6 mm POM fiber on the freeze-thaw damage of PRGRC is better than 12 mm POM fiber. According to the test results, the existing strength attenuation model is further modified, the attenuation model of PRGRC compressive strength under the freeze-thaw cycle is obtained, and the model fitting effect is good. The strengthening mechanism of POM fiber is explained by the structural relationship between POM fiber and concrete matrix in the SEM micrograph of PRGRC. The research results provide a scientific basis for the applicability of POM fiber in geopolymeric cementitious materials and improving the frost resistance of PRGRC.

## 1. Introduction

Polyoxymethylene fiber (POM fiber), polypropylene fiber (PP fiber), basalt fiber, and carbon fiber are commonly used fiber materials to enhance the performance of concrete [1,2,3]. POM fiber has been vigorously promoted in recent years due to its excellent physical and mechanical properties, good wear resistance, strong tensile strength, and excellent durability [4,5,6]. In addition, the unique ether bond in POM fiber can make it better compatible with and dispersed in the concrete matrix [7], which has the effect of toughening and increasing crack resistance while inhibiting shrinkage deformation of concrete. In addition, it can effectively improve the mechanical properties and durability of concrete [8,9]. With the same fiber content, the modification effect of POM fiber is better than that of PP fiber [6]. At the same time, the fiber’s own characteristics determine that the ductility of POM fiber is better than basalt fiber and carbon fiber [10,11].

Geopolymeric concrete (GPC) uses industrial waste such as fly ash and slag as cementitious materials to replace cement. Compared with cement concrete, it can reduce carbon dioxide emissions and the heat energy consumed in production, with obvious advantages in carbon emission reduction [12,13]. In addition, GPC has high early strength, higher strength than ordinary concrete, excellent mechanical properties, good acid and alkali corrosion resistance, outstanding high-temperature resistance, and can solidify heavy metal ions [14,15,16,17,18]. At present, China’s construction waste production is huge, and the recycling of recycled aggregate is a key issue. Some studies show that [19,20,21,22] when recycled aggregate is applied to geopolymeric recycled concrete (GRC), i.e., geopolymeric recycled concrete (GRC), it has higher strength than ordinary recycled concrete. Because the old mortar attached to the surface of recycled aggregate contains alkaline substances, after being added to the geopolymeric concrete, the alkaline substances in the old mortar will promote the polymerization reaction of slag or fly ash, which can effectively improve the interface transition zone and make the GRC matrix denser. However, GPC or GRC will have volume shrinkage deformation in the hardening process [23], which leads to serious concrete cracking. Therefore, considering the advantages of POM fiber in ordinary concrete, it is very practical to try to add POM fiber into GRC as a reinforcing material and explore the effect of adding POM fiber on GRC performance.

In the existing relevant literature, there are many studies on the basic mechanical properties and durability of GRC. Kathirviel Parthiban et al. [24] studied the impact of different recycled aggregate substitution rates on the strength and durability of GRC and found that the performance of GRC with 25% recycled aggregate is equivalent to or even better than that of ordinary concrete. Mahdi Koushkbaghi et al. [25] studied the effect of the ratio of sodium silicate to sodium hydroxide and the replacement ratio of recycled aggregate on the mechanics and durability of geopolymeric concrete. The results showed that with the increase of the sodium silicate/sodium hydroxide ratio, the compressive strength of GRC increased, and the compressive strength of geopolymeric concrete containing RCA decreased by about 8–28% compared with GPC. Duy-Hai Vo et al. [26] studied the mechanical properties and chloride ion corrosion resistance of GRC and found that the higher level of fly ash content is related to the higher chloride penetration resistance. However, there is no report on the frost resistance of GRC at present. The frost resistance of concrete is an important factor affecting the life of concrete structures, especially in cold areas. The pore structure and overall strength of concrete will be greatly affected in cold areas. It is necessary to ensure that concrete can work normally under frozen conditions without failure. Therefore, the frost resistance of concrete is also an important index in practical engineering applications, and there is no research on GRC at present. The frost resistance of concrete is an important factor that affects the life of concrete structures, especially in severe cold areas. Therefore, in this paper, POM fiber is added to GRC to improve its durability. The frost resistance of PRGRC is studied by the freeze-thaw cycle test. The test data and conclusions can fill the gap in the research direction on the frost resistance of GRC. The mass loss, relative dynamic elastic modulus, and compressive strength index of PRGRC reinforced with different lengths and contents of POM fiber reinforced geopolymers under freeze-thaw cycle conditions are measured. The establishment of a numerical model and microstructure analysis reveal the influence mechanism of POM fiber on the frost resistance of PRGRC.

## 2. Materials and Methods

### 2.1. Raw Materials

**Figure 1:**
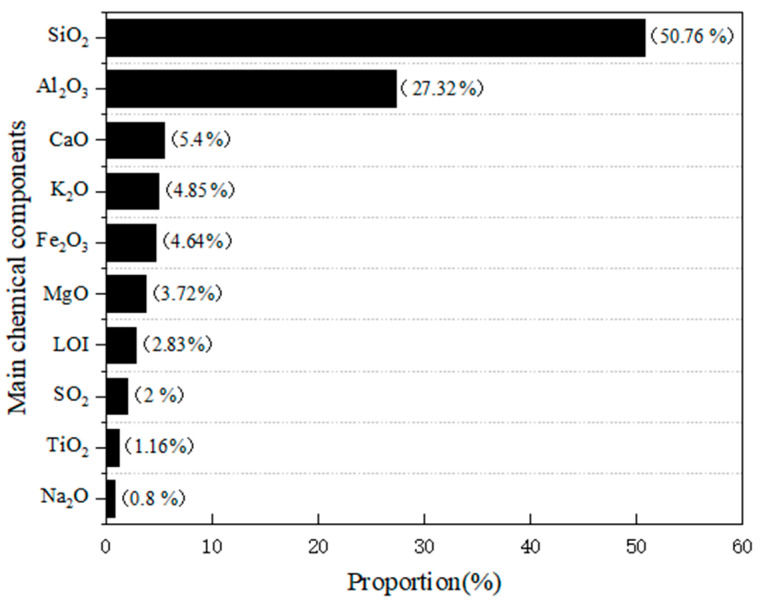
Chemical composition of fly ash (%).

**Figure 2:**
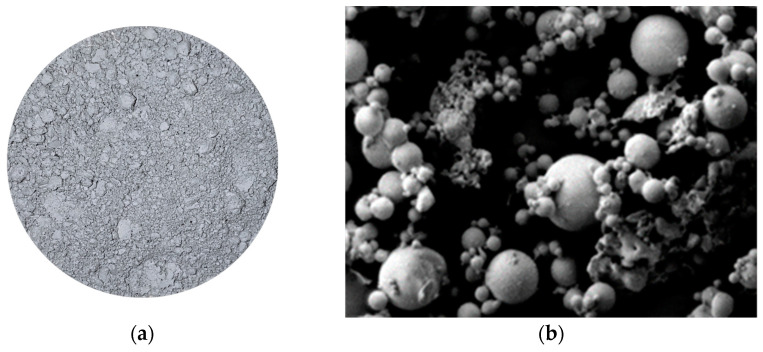
The photo of fly ash: (**a**) Photo of fly ash; (**b**) Micrograph of fly ash.

**Figure 3:**
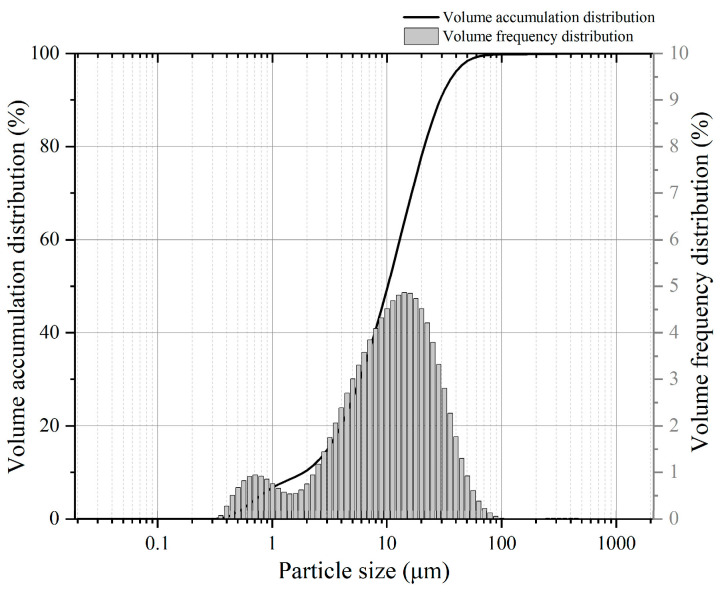
Particle size distribution of fly ash.

The fly ash used in the test is from Tianjin Zhucheng New Material Co., Ltd., Tianjin, China, with an average particle size of 1.201 μm. The main chemical components of fly ash are shown in Figure 1. The material photo and micrograph morphology are shown in Figure 2. The particle size distribution is shown in Figure 3. The fly ash is classified as Class F low calcium fly ash.

**Figure 4:**
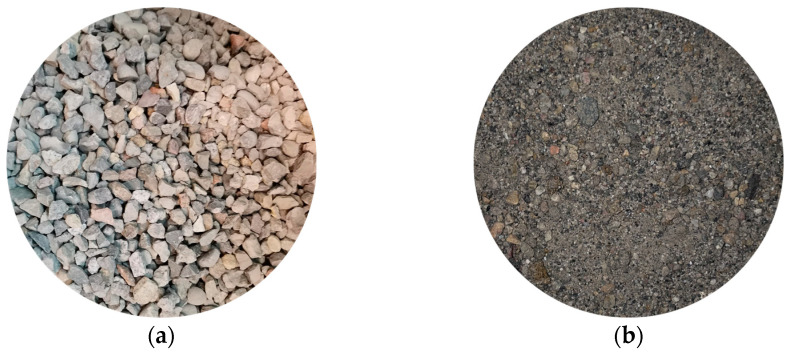
The photo of the aggregate: (**a**) The recycled coarse aggregate; (**b**) The fine aggregate.

The recycled coarse aggregate comes from the concrete after crushing and screening and is selected as 5~20 mm continuous grading. The performance parameters are shown in Table 1. According to the specification for recycled coarse aggregate for concrete (GB/T 25177-2010), it belongs to class II recycled aggregate. The fine aggregate is river sand with a fineness modulus of 2.3, and the maximum particle size is 4 mm. The photo of the recycled coarse aggregate and fine aggregate is shown in Figure 4.

**Figure 5:**
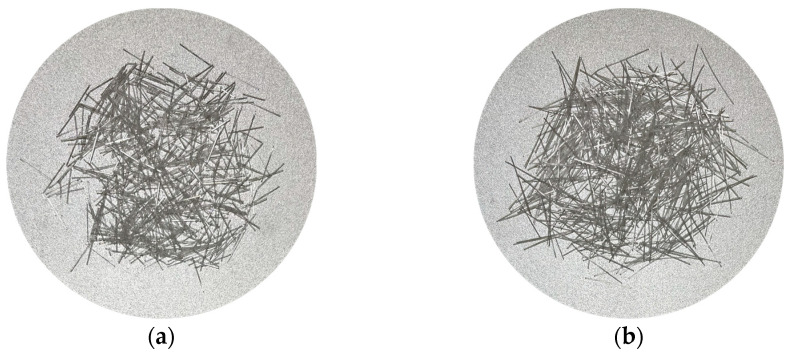
The photo of POM fiber: (**a**) 6 mm POM fiber; (**b**) 12 mm POM fiber.

The density of POM fiber is 1.42 g·cm^−3^, the tensile strength is 800 MPa, the modulus of elasticity is 10 GPa, the elongation at break is 30%, the diameter is 0.2 mm, and the length is 6 mm and 12 mm. The photo of POM fiber is shown in Figure 5. In this test, the mixing volumes of the two lengths of POM fiber are 0%, 0.25%, 0.50%, 0.75%, and 1.00%, respectively. Therefore, the test group number is length-volume; for example, 6-0 represents the length of POM fiber as 6 mm and the corresponding volume content as 0%.

The alkali excitation solution is prepared by sodium hydroxide and sodium silicate solution, and is completed 24 h before use. Sodium hydroxide solution is prepared by dissolving solid sodium hydroxide (NaOH) particles in water, and the concentration is 10 mol/L. The modulus of sodium silicate solution (Ms = m(SiO_2_)/m(Na_2_O)) is 3.13.

The performance of GRC has been determined in previous work. Without adding POM fiber, its strength grade is C50 relative to the strength of ordinary concrete, and its slump is 110 mm.

### 2.2. Preparation of Specimens

Fly ash, coarse and fine aggregates, and alkali activator were proportionally mixed, and POM fiber was added last. The mixed proportion of the test group is shown in Table 2. The concrete was poured into the mold, and the 100 mm × 100 mm × 100 mm mold was selected for the compression test; the 100 mm × 100 mm × 400 mm mold was selected for the dynamic elastic modulus test. The concrete was cured at 80 °C for 24 h before it was demolded, and it was cured at 20 °C for 28 days for the test.

### 2.3. Test Method

Freeze-thaw test refers to the quick-freeze method in the Standard for Long-term Performance and Durability of Ordinary Concrete (GB/T 50082-2009). The test equipment is the KDW-10 concrete rapid freeze-thaw test machine. The freezing temperature was set at −17 °C, and the melting temperature was set at 8 °C. The thermometer was buried in a concrete specimen to react to the temperature change. A freeze-thaw cycle is completed within 2–4 h, and the freeze-thaw transition time does not exceed 10 min. The compressive strength, quality, and transverse fundamental frequency shall be measured after every 25 freeze-thaw cycles. The compressive strength shall be determined according to the Standard for Test Methods of Physical and Mechanical Properties of Concrete (GB/T 50081-2019). Calculate the mass loss rate and relative dynamic elastic modulus according to the measured mass and transverse fundamental frequency, and take the arithmetic mean value of each group of 3 test pieces. The frost resistance grade of PRGRC is determined by the maximum freeze-thaw cycles corresponding to the mass loss rate of the test piece not exceeding 5% and the dynamic modulus of elasticity not less than 60%.

## 3. Results and Discussion

### 3.1. Effect of POM Fiber on Frost Resistance of GRC

#### 3.1.1. Mass Loss Rate

**Figure 6:**
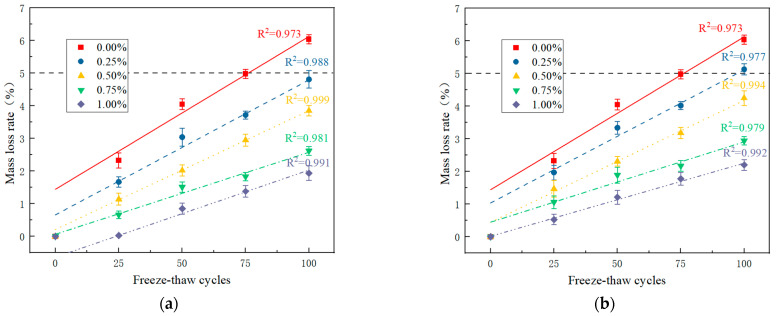
Fitting curve of mass loss rate of PRGRC with the number of freeze-thaw cycles: (**a**) 6 mm POM fiber; (**b**) 12 mm POM fiber.

During the freeze-thaw cycle test of the PRGRC, the hydrostatic pressure and osmotic pressure in the matrix also continue to circulate, and the damage in the specimen continues to accumulate until the outer layer begins to fall off. The mass loss rate of the specimen can be used to analyze the freeze-thaw damage degree of the specimen. The mass loss rate of PRGRC specimens with different POM fiber lengths and volume contents during the freeze-thaw cycle is shown in Figure 6.

It can be seen from Figure 6 that the mass loss rate of PRGRC with 6 mm POM fiber is lower than that of PRGRC with 12 mm POM fiber under the same fiber volume and freeze-thaw cycles. The main reason is that the random distribution of fiber in the concrete with shorter length is better than that of longer fiber. The random distribution of fiber is conducive to strengthening the connectivity of the concrete matrix, thus inhibiting the peeling phenomenon of the concrete surface. Xu, L. [27] and Li, W.J. [28] also found that fiber can reduce the mass loss of concrete under freeze-thaw damage, and the existence of fiber makes the overall connection of the structure higher.

With the increase in freeze-thaw cycles, the rate of mass loss increases. Figure 6a Corresponding to the addition of 6 mm POM fiber, the larger the volume content of the fiber, the slower the increase in the mass loss rate of PRGRC. When the freeze-thaw cycle reaches 100 times, only the mass loss rate of groups of 6-0 specimens exceeds 5%, and the mass loss rate of groups of 6-1.00 specimens is only 1.94%. Figure 6b corresponds to the addition of 12 mm of POM fiber. When the freeze-thaw cycle reaches 100 times, the mass loss rate of the 12-0 and 12-0.25 groups of specimens exceeds 5%, and the mass loss rate of the 12-0.50, 12-0.75, and 12-1.00 groups of specimens with the corresponding frost resistance grade is 4.25%, 2.94%, and 2.20%, respectively. It can be seen from the test results that adding POM fiber can slow down the quality loss of PRGRC during the freeze-thaw cycle. The reason is that the network structure formed after adding POM fiber strengthens the integrity of concrete, improves the adhesion between the base materials, and makes the surface damage degree after freeze-thaw damage low, and adding 1% of 6 mm POM fiber has the best effect on improving the quality damage of the specimen.

#### 3.1.2. Relative Dynamic Elastic Modulus

**Figure 7:**
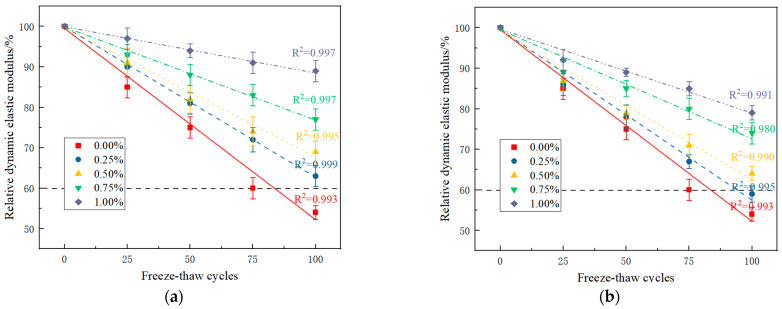
Fitting curve of relative dynamic elastic modulus of PRGRC with the number of freeze-thaw cycles: (**a**) 6 mm POM fiber; (**b**) 12 mm POM fiber.

The internal damage to the PRGRC specimen during the freeze-thaw cycle can be reflected by the change in the relative dynamic elastic modulus. The lower the relative dynamic elastic modulus, the higher the degree of internal damage. The relative dynamic elastic modulus of PRGRC specimens with different POM fiber lengths and volume contents during the freeze-thaw cycle is shown in Figure 7.

The relative dynamic elastic modulus of PRGRC with 6 mm POM fiber is better than that of PRGRC with 12 mm POM fiber (Figure 7). With the increase in freeze-thaw cycles, the relative dynamic elastic modulus of PRGRC with 6 mm POM fiber decreased more slowly than that with 12 mm POM fiber. As fiber content decreases, so does the rate of decrease in the relative dynamic elastic modulus. Before the number of freeze-thaw cycles reached 75, the relative dynamic elastic modulus of all groups was higher than 60%. When the freeze-thaw cycle reaches 100 times, only the relative dynamic elastic modulus of the specimens in groups 6-0, 12-0, and 12-0.25 without fiber is less than 60%; the frost resistance grade is F75, and the maximum is 89% of that in groups 6-1.00. It can be seen that the incorporation of POM fiber can slow down the reduction of the relative dynamic elastic modulus of PRGRC during the freeze-thaw cycle, thus improving the frost resistance of PRGRC. This is also due to the unique ether bond of POM fiber, which makes the fiber and matrix have good compatibility [29]. After the incorporation of fiber, the internal structure of the concrete is improved, the overall compactness is improved, and the effects of toughness and crack resistance are achieved, thus improving the internal damage of the concrete. The POM fiber has a good improvement effect with a length of 6 mm.

#### 3.1.3. Compressive Strength

**Figure 8:**
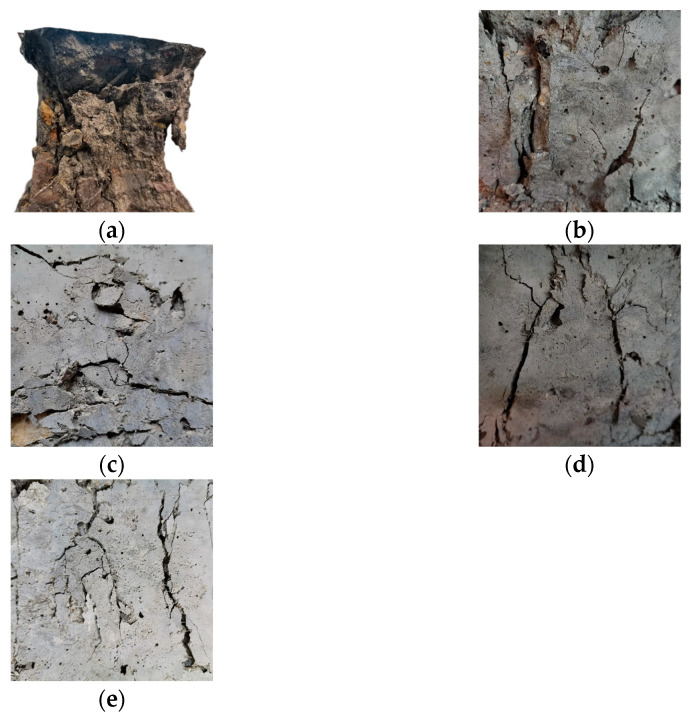
Compressive failure pictures of PRGRC with different fiber volume contents under 100 freeze-thaw cycles: (**a**) 0%; (**b**) 0.25%; (**c**) 0.50%; (**d**) 0.75%; (**e**) 1.00%.

**Figure 9:**
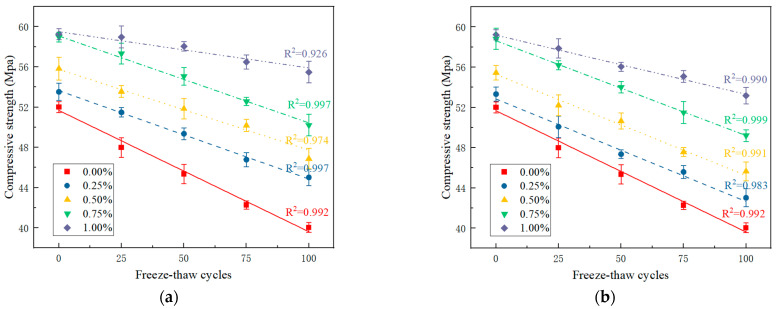
Fitting curve of the compressive strength of PRGRC with the number of freeze-thaw cycles: (**a**) 6 mm POM fiber; (**b**) 12 mm POM fiber.

Internal damage accumulates in PRGRC specimens during freeze-thaw cycles, and the change in mechanical properties is the macroscopic performance. To study the relationship between freeze-thaw damage and the macroscopic mechanical properties of PRGRC and analyze the influence of POM fiber incorporation, the compressive strength test of PRGRC was carried out under different freeze-thaw cycles. Since the damage of the 6 mm POM fiber and the 12 mm POM fiber added to the GRC is similar, the compression failure picture of the PRGRC with the 6 mm POM fiber under 100 freeze-thaw cycles is presented to analyze the PRGRC damage phenomenon. The compression failure picture is shown in Figure 8. The compressive strength of PRGRC specimens with different POM fiber lengths and volume contents during the freeze-thaw cycle is shown in Figure 9.

Figure 8a shows that without adding POM fiber, GRC is severely damaged after compression failure. Due to the brittleness of geopolymeric concrete, obvious peeling occurs around the GRC matrix after compression failure, and the peeling fragments are large, exposing the aggregate in GRC. Figure 8b–e show that the volume content of POM fiber added is 0.25%, 0.50%, 0.75%, and 1.00%, respectively. With the increase of the volume content of POM fiber in the GRC matrix, it can be observed that the peeling degree of PRGRC is significantly reduced, and the integrity of the surface and the whole specimen is gradually improved after the compression failure. When compared to GRC without fiber addition, the improvement of POM fiber on specimen damage is very noticeable. At the same time, after adding POM fiber to GRC, the number of cracks on the surface of PRGRC after damage also decreases with the increase in POM fiber content. Therefore, it can be concluded that the integrity of PRGRC is better than that of GRC after compression failure, which is due to the role of POM fiber in GRC as a caged matrix material to effectively reduce the number of cracks after failure and reduce the degree of cracking.

In Figure 9, the compressive strength of PRGRC with 6 mm POM fiber after freeze-thaw cycles is higher than that of PRGRC with 12 mm POM fiber, and the compressive strength decreases with the increase in freeze-thaw cycles. Compared with GRC without fiber, the fiber has an obvious effect. Li, J.C. [30] also mentioned that fiber can effectively improve the compressive strength and flexural strength of concrete under freeze-thaw cycles, which is related to the improvement of the internal structure and its own characteristics of concrete by fiber. This paper’s findings on the effect of fiber on concrete strength are consistent with current research findings. With the increase in freeze-thaw cycles, the larger the fiber volume fraction, the slower the compressive strength of PRGRC decreases. Without POM fiber, the compressive strength of GRC decreased by 23.03% after 100 freeze-thaw cycles. After adding POM fiber to the GRC, when the fiber volume content changes from 0.25% to 1.00%, the compressive strength of the PRGRC with 6 mm and 12 mm POM fiber decreases from 15.90% and 19.34% to 6.38% and 10.23%, respectively, after 100 freeze-thaw cycles. It is clear that the improvement effect of 6 mm POM fiber on PRGRC compressive strength reduction is superior to that of 12 mm POM fiber. Similarly, Wang, X.C. [31] discovered that the compressive strength of concrete decreased more as the length of carbon fiber in the study increased. Memon, I. [32] discovered in the article that concrete with shorter PP fibers can have relatively high compressive strength. The influence of fiber length on the compressive strength of concrete deserves further study, which is related to the characteristics of fiber and the properties of the concrete materials. POM fiber can improve the strength attenuation of PRGRC after freeze-thaw because, after the POM fiber is added, the strength of the concrete is increased. After all, the internal base material is strengthened by the POM fiber network structure. At the same time, when the concrete is under pressure, the excellent mechanical properties of the fiber itself can also bear part of the pressure. After 100 freeze-thaw cycles, the PRGRC with 1% 6 mm POM fiber has the highest residual strength.

### 3.2. Strength Attenuation Model of PRGRC

According to previous studies [33], the strength and freeze-thaw times of concrete under freeze-thaw cycles conform to the following model laws

(1) $$\begin{array}{c} \frac{f^{D}}{f}=A\exp\left(\frac{N}{B}\right) \end{array}$$

where *f* is the strength of the concrete specimen before freezing and thawing, *N* is the number of freezing and thawing cycles, *f^D^* is the corresponding concrete strength when the number of freezing and thawing cycles is *N*, and the constants *A* and *B* are related to the material and temperature.

However, this model does not meet the boundary condition of *A* = 1 when *N* = 0, and it cannot accurately simulate the change in concrete compressive strength with the number of freeze-thaw cycles under the freeze-thaw cycle conditions. Therefore, in combination with the Weibull distribution probability density function, the model can be modified as follows

(2) $$\begin{array}{c} \frac{f^{D}}{f}=\exp\left[-{\left(\frac{N}{B}\right)}^{A}\right] \end{array}$$

Taking the logarithm of both sides of the equal sign of Equation (2) twice to get

(3) $$\begin{array}{c} \ln\left[\ln\left(\frac{f}{f^{D}}\right)\right]=A\ln N+A\ln\left(\frac{1}{B}\right) \end{array}$$

When $\ln\left[\ln\left(\frac{f}{f^{D}}\right)\right]=Y,\;\;\ln N=X$

Equation (3) can be written as a first-order linear function

(4) $$\begin{array}{c} Y=AX+A\ln\left(\frac{1}{B}\right) \end{array}$$

**Figure 10:**
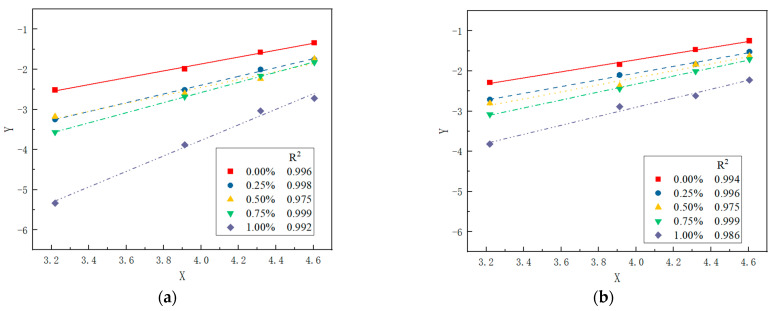
Fitting curve of compressive strength and freeze-thaw cycles of PRGRC in the strength attenuation model: (**a**) 6 mm POM fiber; (**b**) 12 mm POM fiber.

According to the test data of the compressive strength of PRGRC with different fiber lengths and volume contents under the freeze-thaw cycle, Equation (4) is linearly fitted, as shown in Figure 10. The linear regression parameters are shown in Table 3, and the correlation coefficient R^2^ is above 0.97, which indicates that the compressive strength of PRGRC with different POM fiber lengths and volume contents under the freeze-thaw cycle is highly fitted with the model.

**Figure 11:**
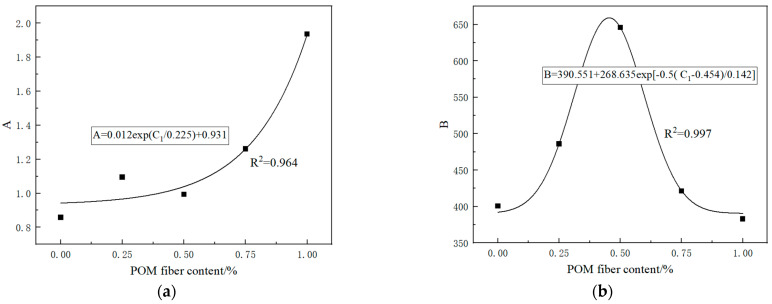
Fitting curve between volume content of 6 mm POM fiber and parameters *A* and *B*: (**a**) Parameter *A*; (**b**) Parameter *B*.

**Figure 12:**
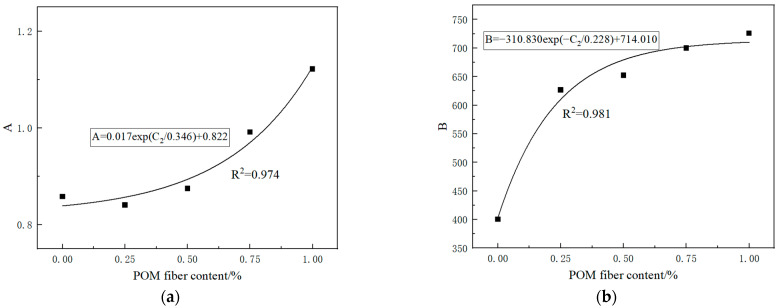
Fitting curve between volume content of 12 mm POM fiber and parameters *A* and *B*: (**a**) Parameter *A*; (**b**) Parameter *B*.

The equation for the compressive strength decay of PRGRC with different POM fiber length and volume content under freeze-thaw cycle conditions can be obtained by substituting the relevant parameters obtained by fitting them into Equation (2). Among them, the values of parameters A and B are related to the volume content of POM fiber. The volume content of POM fiber is taken as an independent variable, and the parameters A and B are taken as dependent variables for fitting. The fitting results are shown in Figure 11 and Figure 12.

The relationship between parameters *A* and *B* and the volume content *C*_1_ of 6 mm POM fiber is as follows

(5) $$\begin{array}{c} A=0.012\exp\left(\frac{C_{1}}{0.225}\right)+0.931 \end{array}$$

(6) $$\begin{array}{c} B=390.551+268.635\exp\left[-\frac{0.5\left(C_{1}-0.454\right)}{0.142}\right] \end{array}$$

For 12 mm POM fiber, the relationship between parameters *A* and *B* and its volume content *C*_2_ is as follows

(7) $$\begin{array}{c} A=0.017\exp\left(\frac{C_{2}}{0.346}\right)+0.822 \end{array}$$

(8) $$\begin{array}{c} B=-310.830\exp\left(-\frac{C_{2}}{0.228}\right)+714.010 \end{array}$$

Substitute Formulas (5)–(8) into Formula (2) to obtain the PRGRC compressive strength attenuation model with 6 mm and 12 mm POM fiber. The model has a good fitting effect and can effectively reflect the change rule for the compressive strength of PRGRC after freeze-thaw damage.

### 3.3. Strengthening Mechanism of POM Fiber

As for the freeze-thaw damage theory of concrete, the hydrostatic pressure theory and osmotic pressure theory proposed by Powers and Helmuth are widely accepted [28]. This theory also applies to PRGRC freeze-thaw damage. During the freeze-thaw cycle, under the effect of temperature cycling, the hydrostatic pressure and osmotic pressure cycling in the PRGRC matrix lead to the continuous accumulation and development of the initial damage in the concrete. The cyclic change process of hydrostatic pressure and seepage pressure under constant loading and unloading is similar to the fatigue failure of concrete structures, so the freeze-thaw damage of PRGRC can be regarded as a fatigue damage effect [34].

**Figure 13:**
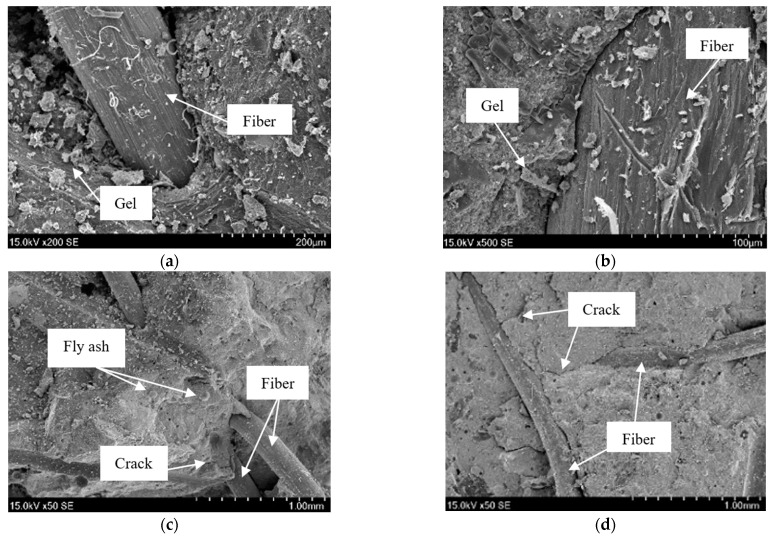
SEM micrograph of the connection between POM fiber and GRC matrix: (**a**) Single 6 mm POM fiber; (**b**) Single 12 mm POM fiber; (**c**) Multiple 6 mm POM fibers; (**d**) Multiple 12 mm POM fibers.

Figure 13 is the microstructure of PRGRC under the scanning electron microscope (SEM), which can effectively observe the fiber interfacial transition zone (FITZ) between fiber and concrete so as to analyze the interaction between POM fiber and GRC, including the embedded distribution of fiber, the control effect of fiber on the concrete cracks, etc. [35]. From Figure 13a,b, it can be seen that the connection between a single POM fiber and concrete is good, and the POM fiber is tightly embedded in the concrete matrix, indicating that the POM fiber and concrete have good compatibility, which is conducive to the improvement of the performance of POM fiber on concrete matrix. From Figure 13c,d, it can be seen that many POM fibers form a network structure in the concrete matrix, which plays a role in the densification and reinforcement of the concrete matrix structure while inhibiting the shrinkage effect of the concrete itself, thus improving the strength and providing strong support for the subsequent concrete matrix to reduce damage in the freeze-thaw cycle.

During the freeze-thaw cycle, when the damage in the PRGRC matrix has occurred and begun to spread around, the POM fibers uniformly distributed in the matrix into a network structure play a role in connecting and supporting and can consume a part of the tensile stress, thus slowing down the growth rate of freeze-thaw damage and having a good anti-crack effect. In addition, the initial defects in the PRGRC matrix include the number and size of pores and the length of microcracks, which are also the main reasons for determining the frost resistance of concrete [36].

After adding POM fiber to the GRC, the POM fiber will make the internal structure of the PRGRC matrix loose and make the pores in the PRGRC matrix have a certain expansion space to withstand a part of the hydrostatic pressure and seepage pressure. At the same time, POM fiber itself can also withstand some water pressure. Therefore, the incorporation of POM fiber can effectively reduce the hydrostatic pressure and seepage pressure of the PRGRC matrix under freeze-thaw cycles and inhibit the expansion of pores and cracks. On the other hand, the addition of POM fiber will increase the air content in the PRGRC matrix, increase the beneficial pores, and also block a part of the pores, making the water in them unable to circulate with each other, weakening the damage caused by the freeze-thaw cycle.

However, if too much POM fiber is added to the GRC, the fluidity of the PRGRC will deteriorate during the mixing and pouring process, and the POM fiber cannot be completely wrapped in the slurry, so more initial defects will be produced in the PRGRC and the mechanical properties and frost resistance will be reduced. Therefore, the reasonable length and content of POM fiber can maximize the strengthening and toughening effect of POM fiber on PRGRC.

## 4. Conclusions

In this paper, the frost resistance of PRGRC mixed with POM fiber is studied through the freeze-thaw cycle test. Based on the analysis of the data obtained from the test, the following conclusions can be drawn:

(1) POM fiber can effectively reduce the damage of PRGRC under freeze-thaw cycles. With the increase of POM volume, the adverse change trend of mass loss rate, relative dynamic elastic modulus, and compressive strength are improved, and the frost resistance of PRGRC is improved. The improvement effect of 6 mm-long POM fiber on the freeze-thaw damage of PRGRC is better than that of 12 mm-long POM fiber.
(2) The compressive strength attenuation model of PRGRC under freeze-thaw cycles based on the test results can provide a theoretical basis for the subsequent research on POM fiber-reinforced geopolymeric recycled concrete.
(3) The reasons why POM fiber in PRGRC can improve the freeze-thaw damage effect are summarized as follows: the first is that the POM fiber plays a supporting role in the network structure of the PRGRC matrix, which slows down the rate of matrix damage under freezing and thawing conditions. At the same time, the POM fiber loosens the internal structure of the matrix, making the internal pores bear a part of hydrostatic pressure and osmotic pressure. The second reason is that POM fiber bears a part of the pressure depending on its own material characteristics. The optimum content of POM fiber under the test conditions is 1%.

This paper only studies the influence of POM fiber on the frost resistance of GRC. Later, we can expand the research on the early shrinkage and sulfate resistance of POM fiber on GRC with different length and volume and combine different test methods, such as nuclear magnetic resonance, nanoindentation on its pore structure, and aggregate-cement interfacial transition zone (ITZ), to further analyze the impact of POM fiber on the performance of GRC. The research in the field of GRC needs to be supplemented.

## Figures and Tables

**Table 1:** Performance parameters of coarse aggregate.

Aggregate Type	Natural Aggregate	Recycled Aggregate
Apparent density/(kg/m^3^)	2632	2566
Bulk density/(kg/m^3^)	1479	1435
Water absorption/%	0.53	5.52
Crushing index/%	5.83	13.03

**Table 2:** Mix proportion design of PRGRC.

Material	Mix Proportions (kg·m^−3^)
Fly ash	460
Recycled coarse aggregate	1200
Fine aggregate	540
Na_2_SiO_3_	133.4
NaOH	20.94
H_2_O	45.76
POM	0/3.55/7.10/10.65/14.20

**Table 3:** Linear regression parameters of strength attenuation.

Test Number	A	B
6-0/12-0	0.8580	400.5550
6-0.25	1.0952	486.0285
6-0.50	0.9929	645.8980
6-0.75	1.2612	421.3779
6-1.00	1.9356	382.9572
12-0.25	0.8405	626.8392
12-0.50	0.8746	652.3378
12-0.75	0.9914	699.7908
12-1.00	1.1218	725.8618

## Data Availability

Data are contained within the article or are available on request from the corresponding author.
